# A Clinical Lecture on Stone in the Ureter

**Published:** 1910-10-01

**Authors:** J. W. Thomson Walker

**Affiliations:** Assistant Surgeon to St. Peter's Hospital for Stone.


### Hospital Clinics.

### A CLINICAL LECTURE ON STONE IN THE URETER.

By J. W. THOMSON WALKER, F.R.C.S., Assistant Surgeon to St. Peter's Hospital for Stone.

(Delivered at the Polyclinic, Chenies Street, W.C.)

Gentlemen,—I have brought before you to-day eight cases of stone in the ureter which have recently come under my observation. The stones in these cases lay at the level of the brim of the bony pelvis or below this. These cases illustrate the growth in size of a calculus in the ureter and the destructive effects it produces on the kidney on the same side, and they raise certain practical points which are of interest to you. These stones are formed in the pelvis of the kidney, and when they commence their journey down the ureter they are of small size. Many of the stones which are formed in the pelvis of the kidney remain there and form kidney stones; and some of these, even when they are small, do not, apparently, make any attempt to descend. The shape of the stone appears to have some influence in deciding whether it will descend or not, for the great majority of single stones that pass through the ureter are shaped like a very small date-seed. A smooth surface is also conducive to a successful transit, but only if the stone is very small.

### Points of Impaction.

There are three points at which the migrating stone may be arrested; namely, at a point just below the level of the lower pole of the kidney, at the brim of the bony pelvis, and just outside the bladder. At these points the ureter is narrower than elsewhere.

These do not, however, exhaust the positions at which a stone may be arrested. I have removed stones from the ureter above the brim of the pelvis and from points between the brim of the pelvis and the entrance of the ureter into the bladder. They are especially liable to be arrested at the point where the ureter turns forwards to pass along the side of the rectum. They may also be found actually inside the bladder muscle, but still impacted in the part of the ureter which underlies the mucous membrane.

When a stone becomes fixed in the ureter it slowly increases in size. Sometimes the new material is deposited all round the stone, so that it becomes round in shape; but usually the deposit takes place at the end which is nearest the kidney, and a long calculus is formed, at the lower end of which the original calculus can be seen. Two of the cases that I have shown to-day display very clearly this method of growth. Two or more stones may be found. In seven of these cases there was one stone; in one there were two.

### Symptoms.

The symptoms caused by stone in the ureter vary considerably. Renal colic is the most frequent. The pain usually commences at the renal area, in the angle between the erector spinae mass of muscle and the last rib, and passes through to the front of the abdomen and down along the line of the ureter into the testicle or along the urethra.

After the attacks of colic there is frequently a fixed aching pain situated over the point at which the calculus is arrested. The aching or discomfort is worse after exercise and on taking large quantities of fluid.

Hæmaturia may accompany the attacks of colic. It may be the only symptom, and occur quite apart from any pain; it may, on the other hand, be entirely absent. In the early stage there is no pus in the urine. In one of my cases there was transient bacilluria. In the later stages there is invariably pyuria. One patient whose case I have described had polyuria, amounting to 120 ounces or more of urine in twenty-four hours, and this fell to 50 ounces after I removed the stone. Sometimes there is frequent micturition and pain on micturition when the stone is low down in the ureter.

When the stone is in the pelvic portion of the ureter it can be felt by the finger in the rectum unless it is very small. The diagnosis is made by means of the *x*-rays, and both kidneys and the whole length of both ureters should be searched by this means. Difficulties in diagnosis by the *x*-rays may be caused by the presence of shadows due to calcareous glands, to phleboliths, to calcareous deposits in blood-vessels, to opaque bodies in the bowel, and, where there has been a previous operation, to thickening around or calcareous deposit upon silk ligatures. In one of the cases that I have shown to-day the patient had been treated at another hospital for dyspepsia, and had been given medicine containing bismuth. The *x*-rays showed large irregular opacities in the abdomen due to the bismuth in the intestine. In spite of constant purging, a clear radiograph was only obtained 4½ months after the bismuth had been stopped.

### X-ray Proofs.

When a small shadow in a radiograph is suspected to be due to a stone in the ureter, the points which should guide you in making the diagnosis are the position of the shadow in the line of the ureter and the shape of the stone. An oval shadow is very typical of small ureteric stone. At the same time the shadow thrown by a ureteric stone may be round, and the shadow may appear to lie away from the line of the ureter.

It is very important to have the patient placed in a definite and constant position in regard to the electric spark and the photographic plate, for you will realise that a radiogram taken with the rays falling obliquely on the plate will make it difficult or impossible to localise accurately the position of the shadow. A radiographer experienced in photographing ureteric calculi will adopt methods to overcome this difficulty, and will be able to reproduce the exact relation of patient, *x*-rays, and plate at any subsequent examination, and thus to give an opinion as to whether the shadow has changed its position or not.

Even with an accurately taken radiograph it is sometimes difficult to say whether a small shadow is in the line of the ureter or not, and the symptoms may not give much assistance. We must then resort to another method of diagnosis; namely, the passage of a bougie up the ureter. The ureter may thus be sounded for stone. This is carried out by means of a special cystoscope, along which a small tunnel runs, and through this the bougie is passed so that the point can be seen in the cystoscopic field. This is guided into the ureteric orifice and the bougie passed gently up the ureter. It is impossible to use a metal sound, as one might do in the urethra, for the bougie must be softer and more flexible than metal can be made; and, moreover, the grating of the metal bougie against the metal of the cystoscope would prevent any metallic click given by the stone being appreciated by the fingers of the operator. Soft bougies are therefore used. It has been recommended that wax-tipped bougies should be used, so that the rough surface of the stone will scrape the wax and the scratches can be seen after withdrawing it. This may sometimes be possible in the female subject, when a large Kelly's tube and reflected light may be used, but it is not practicable in the male subject, when the cystoscope must be used; nor does it give us any information that we cannot obtain by other means.

### Bougies and Cystoscopy.

On passing a solid bougie up the ureter it will be arrested by the stone, if one is present. There are, however, other causes, such as folds of mucous membrane, kinks of the ureter, etc., which may arrest the passage of a bougie, so that this is not absolutely diagnostic. The most certain method is to pass a bougie which is opaque to the $x$-rays and have a radiogram made with the bougie in place.

You will see from the radiograms that I have shown you that this has been done in three of my cases, where the position and shape of the shadow had left the diagnosis doubtful.

The bougie can be traced through the bladder and along the ureter, and at the tip of it is seen the shadow of the stone. In one of my cases, when a large smooth stone lay in the ureter below the brim of the pelvis, the bougie, after a slight hitch, passed alongside it and on to the kidney. The radiogram shows the shadow cast by the stone and the bougie passing alongside it. It is well to remember that a small pure uric acid stone does not throw a shadow with the $x$-rays.

Cystoscopy in cases of ureteric calculus may show œdema of the ureteric orifice or a dilated or ecchymosed orifice, but very frequently there is nothing abnormal to be seen.

### Treatment.

There are two methods of treatment of ureteric stones—the diuretic and the operative. In large stones there is no choice between these methods, for an operation is the only means of getting rid of the stones, but when the stones are small the merits of the two methods will have to be discussed. When a patient has previously passed a stone and a small ureteric stone is diagnosed, it is probable, but not certain, that he will pass the second stone. The stones that are most likely to pass are those with an oval shape and smooth surface, but many stones are passed which are covered with irregular sharp crystals. Larger stones, and especially those which have become round from fresh deposit of salts, will rarely pass. The diuretic treatment aims at assisting the expulsion of the stone by increasing the volume of fluid passed through the ureter. It is impossible by any means at present known to dissolve stones in the ureter, and you may disregard any statements to the contrary.

There are many diuretic waters and drugs, which I need not discuss in detail. I use large quantities of distilled water, Contrexéville water, and such salts as citrate of potash. A visit to Contrexéville or Vittel will sometimes be successful when diuretic treatment in this country has failed.

A limit should be placed on the length of time during which the diuretic treatment is continued. A stone which is fixed in the ureter gradually increases in size and destroys the kidney, partly by blocking the ureter and partly by the inflammation which it eventually sets up.

Chronic pyelonephritis, hydronephrosis, and pyonephrosis are the forms of renal disease that are produced.

The symptoms of this slow destruction of the kidney are quite insignificant, and there may be no renal symptoms until the kidney has been almost destroyed.

Slight renal aching and tenderness and a trace of pus and some tube-casts in the urine may be all, until the kidney is distended and destroyed by an accumulation of urine or pus, and can be detected by palpation. I usually limit the period for diuretic treatment to a year, and, should there be any sign of renal disease or of urinary infection before this time is up, insist on immediate operation.

### Operation.

The best method of operation for all stones situated at or below the brim of the pelvis is by an incision running parallel to and two inches above Poupart's ligament, extending inwards to the edge of the rectus sheath and outwards to the level of the anterior superior iliac spine of the ilium. The abdominal muscles are cut through, and care must be taken at the inner end of the wound not to injure the vessels of the cord. The transversalis facia is incised and the peritoneum raised up. The patient at this stage is placed in the Trendelenberg position. The peritoneum is stripped up along the line of the external iliac artery, and the ureter is found on the peritoneum about the position of the bifurcation of the common iliac artery. If the stone lies at the pelvic brim it will readily be felt and removed by means of a longitudinal incision. If it lies lower down in the pelvic portion of the ureter, the duct should be traced down with the finger and the hard nodule felt. If the stone is small, the ureter should be opened above the line of the iliac artery, and a fine flexible metal scoop passed down the duct. With this inside the ureter and the finger outside, the stone is easily pulled up to the opening and removed. This may be carried out whether the ureter is dilated or not. If the ureter is dilated, a stone of considerable size may be drawn up in this way and removed. This is facilitated by the use of long fine forceps which I have introduced. If, however, the stone is large and fixed, it will be necessary to cut down upon it direct, making the incision in the long axis of the stone. The ureter in these cases is surrounded by adhesions, and care must be exercised not to injure the large blood-vessels of the pelvis which lie in the immediate neighbourhood.

After removing the stone a bougie should be passed down the ureter into the bladder to make certain that no stricture exists. The wound in the ureter is then closed by interrupted catgut sutures. The sutures must not consist of silk, for fresh stones will form on the sutures. In one of my earliest cases of ureteric calculus I stitched up the ureteral wound with fine silk, avoiding, as I thought, the mucous membrane of the ureter. The patient afterwards passed two stones in which were embedded the silk sutures.

In one case, in which about a pint of pus was removed from the kidney and ureter through the incision made to remove the stone, I left a part of the wound in the ureter open and allowed it to drain for a few days, and it rapidly closed and the wound healed up. In the other cases I closed the wound in the ureter completely and placed a drain in the iliac fossa. A little urine sometimes leaks out for two or three days, and after this the wound is dry and the tube may be removed.

It is important that the tube should be placed in the iliac fossa, well above the iliac artery. I know of a case in which a rubber tube left in contact with the iliac artery gave rise to sloughing of the arterial wall and fatal hæmorrhage.

If the incision in the ureter is below the pelvic brim I tilt the foot of the bed so that any urine which may leak gravitates into the iliac fossa.

The patient is kept in bed for three weeks to allow the abdominal wound to heal firmly.

